# Comprehensive proteomic analysis of exoproteins expressed by ERIC I, II, III and IV *Paenibacillus larvae* genotypes reveals a wide range of virulence factors

**DOI:** 10.1080/21505594.2019.1603133

**Published:** 2019-04-18

**Authors:** Tomas Erban, Justyna Zitek, Miroslava Bodrinova, Pavel Talacko, Milan Bartos, Jaroslav Hrabak

**Affiliations:** a Proteomics and Metabolomics Laboratory, Crop Research Institute, Prague, Czechia; b Department of Parasitology, Faculty of Science, Charles University, Prague 2, Czechia; c Proteomics Core Facility, Faculty of Science, Charles University, BIOCEV, Vestec, Czechia; d BioVendor – Laboratorni medicina a.s., Brno, Czechia; e Laboratory of Antibiotic Resistance and Applications of Mass Spectrometry in Microbiology, Biomedical Center and Institute of Microbiology, Faculty of Medicine in Plzen, Charles University, Plzen, Czechia

**Keywords:** American foulbrood, Apis mellifera, immune inhibitor A, mosquitocidal toxin, microbial collagenase ColA, Bacillus thuringiensis, bacteriocin, S-layer protein, ADP-ribosylating toxin, polysaccharide deacetylase

## Abstract

American foulbrood is a quarantine disease of the honeybee *Apis mellifera* L. in many countries and contributes greatly to colony losses. We performed a label-free proteomics study of exoprotein fractions produced *in vitro* by *Paenibacillus larvae* reference strains of the ERIC I–IV genotypes. A quantitative comparison was performed of previous studied protein-based virulence factors and many newly identified putative virulence factors. Among the multiple proteases identified, key virulence factors included the microbial collagenase ColA and immune inhibitor A (InhA, an analog of the *Bacillus thuringiensis* protein InhA). Both of these virulence factors were detected in ERICs II–IV but were absent from ERIC I. Furthermore, the different S-layer proteins and polysaccharide deacetylases prevailed in ERICs II–IV. Thus, the expression patterns of these virulence factors corresponded with the different speeds at which honeybee larvae are known to be killed by ERICs II–IV compared to ERIC I. In addition, putative novel toxin-like proteins were identified, including vegetative insecticidal protein Vip1, a mosquitocidal toxin, and epsilon-toxin type B, which exhibit similarity to homologs present in *Bacillus thuringiensis* or *Lysinibacillus sphaericus*. Furthermore, a putative bacteriocin similar to Lactococcin 972 was identified in all assayed genotypes. It appears that *P. larvae* shares virulence factors similar to those of the *Bacillus cereus* group. Overall, the results provide novel information regarding *P. larvae* virulence potential, and a comprehensive exoprotein comparison of all four ERICs was performed for the first time. The identification of novel virulence factors can explain differences in the virulence of isolates.

## Introduction

*Paenibacillus* is an important bacterial genus with members that can be isolated from diverse environments, and they play important roles in soil, animals and plants. Many *Paenibacillus* species have been found to produce biomolecules of scientific interest with potential industrial use. Thus, the genus *Paenibacillus* has recently been the subject of scientific reviews [1–3]. In an attempt to generate additional data on the relatively poorly understood *Paenibacillus* taxon, detailed studies have been performed to identify and characterize *Paenibacillus* isolates to facilitate their utilization [4,5]. In addition to the diverse beneficial *Paenibacillus* species, important pathogens of beneficial insects are also members of this genus [1,6]. The most important insect-pathogenic member is *Paenibacillus larvae* [7], which is the causative agent of American foulbrood (AFB), a brood disease that causes substantial honeybee colony losses worldwide[8].

Similar to other pathogens, AFB development is attributed to specific virulence factors. Heyndrickx *et al.* [9] reclassified *P. larvae* into two subspecies, *P. larvae* subsp. *larvae* (*P. l. larvae*) and *P. larvae* subsp. *pulvifaciens* (*P. l. pulvifaciens*), based on phenotypic and genotypic differences and AFB pathology differences [9]. Previously, *P. larvae* and *P. pulvifaciens* were proposed based on 16S rRNA analysis [7]; however, this classification was not adequately justified [10]. The repetitive element PCR fingerprinting (rep-PCR) technique using specific primers for the loci of BOX A1R, MBO REP1 and ERIC [11] was used to study German isolates of *P. l. larvae *[12]. Subsequent studies showed differences in the biochemical properties of isolates [13] as well as differences in their ability to induce honeybee mortality [14]. In 2006, Genersch *et al.* [15] proposed the classification of *P. larvae* into four genotypes (ERIC I–IV) using ERIC-PCR, and since then, this classification scheme has been accepted by the scientific community. Furthermore, a decade later, the use of various techniques, such as MALDI-TOF mass spectrometry (MS) analysis of whole cells [16], multi-locus sequence typing (MLST) [17] and multi-locus variable number of tandem repeat analysis (MLVA) as an alternative to MLST [18], confirmed that categorization into the ERIC genotypes is appropriate despite the fact that observed heterogeneity in isolates indicates subtypes [16–19].

The former subspecies *P. l. larvae* corresponds to the genotypes ERIC I and II, while the former subspecies *P. l. pulvifaciens* corresponds to the genotypes ERIC III and IV [8]. The most common genotype worldwide is ERIC I, followed by ERIC II, while genotypes III and IV have a low prevalence [16–19]. The ERIC I genotype is considered to be less virulent than the other ERIC genotypes since it is able to kill 100% of larvae approximately 12 to 14 days after inoculation, whereas the other three ERIC genotypes kill all the larvae within 6 to 7 days [20–22]. Understanding the mechanisms underlying the virulence of different strains based on their specific genotype is key to predicting the efficiency with which *P. larvae* kills the host (including the whole colony) [14,20,21,23].

Virulence factors of *P. larvae* suggested based on genome analyses primarily include various proteases, collagenases, chitinases, S-layer proteins, toxins (i.e. ETX/MTX2, VIP2, Binary toxin B), secondary metabolites [14,20,21,23], and potentially other biologically active substances. In particular, another suggested virulence factor is enolase [24], and metalloprotease activity was detected in several studies investigating these enzymes as putative virulence factors responsible for tissue lysis [25–27]. Subsequent functional studies indicated the importance of the following virulence factors: S-layer protein SplA [28], toxins Plx1 and Plx2 [29], chitin-degrading protein *Pl*CBP49[30], and ADP-ribosyltransferase C3larvin toxin [31], and Plx2A has been shown to be a C3-like toxin [32]. Furthermore, inhibition by a protease of the insect immune-associated cecropin-like activity has been observed in insect hemolymph [33]. Finally, in addition to the above-listed protein-based virulence factors, various secondary metabolites have been studied as other potential virulence factors [34–39].

Until now, most of the studies related to *P. larvae* virulence have been performed on ERIC I and II genotypes due to their prevalence. However, understanding the virulence mechanisms of the former *P. l. pulvifaciens* (ERIC III and IV genotypes of *P. larvae*) is also essential because different virulent strains can emerge as observed for other pathogens throughout human history. Thus, in this study, we sought to identify the exoproteins of all four ERIC genotypes. To this end, we utilized a label-free quantitative (LFQ) proteomic approach employing nano-liquid chromatography coupled with a state-of-the-art Orbitrap Fusion^TM^ Tribrid^TM^ mass spectrometer (nanoLC-MS/MS) (Thermo Fisher Scientific, Waltham, MA, USA). A comparison of the proteome profiles of protein fractions from media filtered from cells of the ERIC I–IV reference strains showed important differences among the genotypes. Our results provide new information regarding the existing protein-based virulence factors and identify potential novel contributors to *Paenibacillus* virulence.

## Results and discussion

In this study, we performed a comprehensive proteomic analysis of the exoprotein fraction of four reference *P. larvae* ERIC I–IV genotypes. The exoprotein fraction can be characterized as the liquid medium used for cultivation eliminated from the bacterial cells. Thus, it should be enriched in secreted extracellular and surface-associated proteins; however, in the data evaluation, it is appropriate to consider that some proteins can come from the intracellular region due to the disruption of bacteria.

**Figure 1.**
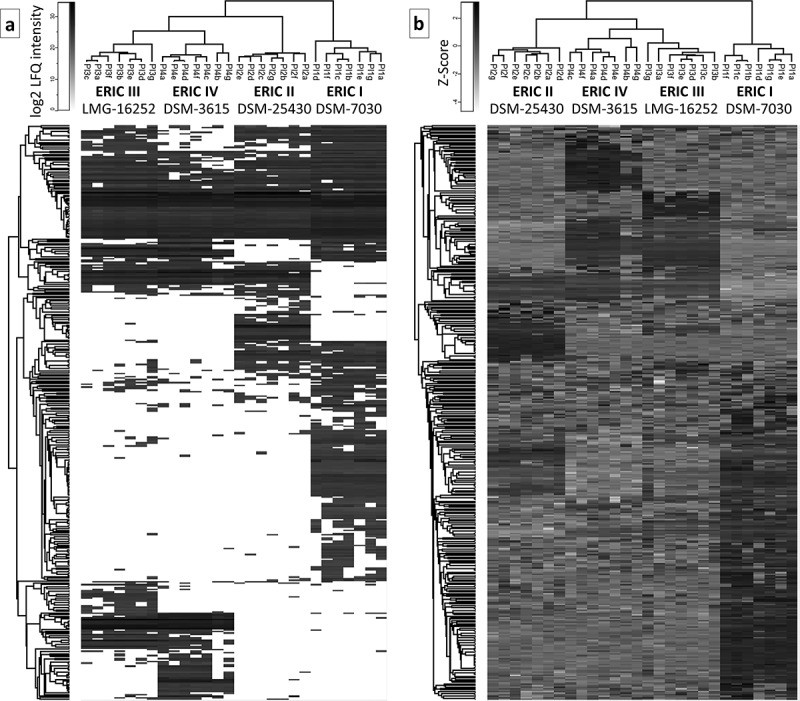
Two heatmaps that visualize the proteome differences in exoproteins of the four genotypes. **a)** Heatmap generated after the missing values were replaced by the constant “0.” The presentation demonstrates that the number of proteins was not detected in all 7 biological replicates (denoted with a–g) within the genotype. **b)** Heatmap was generated after the missing values were replaced from a normal distribution, and the data were subtracted using a Z-score. Both data evaluation methods provide similar results in which ERIC I clusters separately from ERICs II–IV, which likely corresponds to the difference in the efficiency at which these genotypes kill honeybee larva. Overall, the results indicate a similarity between ERIC III and IV and a substantial difference between ERIC I and II.

**Figure 2.**
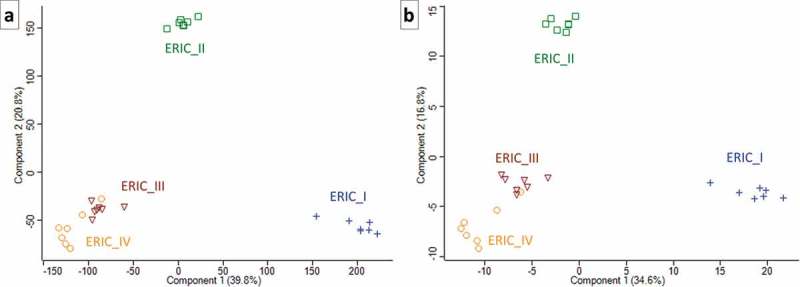
Two PCA charts that illustrate the proteome differences in exoproteins of the four genotypes. Each of the symbols represents one biological replicate, and the analysis clearly demonstrates the homogeneity of the samples or analyses. The same numerals used to generate the heatmaps in Figure. 1(a) and Figure 1(b) were used to produce the charts in Figures 2(a,b), respectively. The similarity in ERIC III and IV and separate positions of ERIC II and ERIC I are clearly demonstrated.

Twenty-eight *P. larvae* samples representing all four genotypes were prepared as seven biological replicates and consecutively analyzed by nanoLC-MS/MS. Because the bacteria were cultivated in medium containing yeast extract and Mueller-Hinton broth (see Materials and methods), specific steps were necessary to prevent inaccurate identifications due to the yeast and bovine proteins. Therefore, we included the yeast and bovine protein sequences in the list of contaminants in MaxQuant [40]. This step enabled the raw dataset of 1,160 protein results (excluding reverse and only identified by site hits) to be easily and rapidly distinguished from the 478 hits, which were potential contaminants mostly of the yeast and bovine origin. Thus, the high-throughput LFQ proteomic approach yielded a dataset of 682 hits corresponding to *P. larvae* (Table S1). Further, it is appropriate to consider traces in the analysis, especially because we used the highly sensitive and high-resolution Orbitrap Fusion^TM^ Tribrid^TM^ MS instrumentation that combines quadrupole, Orbitrap and ion trap (q-OT-IT). The traces should be eliminated through the next step. After data filtering to at least 3 valid values in at least one variant (genotype), 301 hits were discarded, which resulted in a representative dataset of 381 hits (Table S1) that were further used to create the heatmaps (Figure 1) and applied in the principal component analysis (PCA) (Figure 2). However, the 301 removed hits are included in Table S1 (marked in red and in column AC denoted as Discard) to make these results available for consideration; moreover, the identified proteins that presented low abundance may be of interest because their expression can be context specific.

The dataset consisting of 381 hits was evaluated using two algorithms in Perseus [41] and used to generate two heatmaps to allow for visual comparison and hierarchical clustering. One heatmap (Figure 1(a)) was generated after the missing values were replaced by the constant “0”. This method of data evaluation nicely demonstrated the presence of “undetected” proteins in certain strains of *P. larvae*. The complete absence of identified proteins in all 7 biological replicates indicates that the genotype did not produce an exoprotein or produced a negligible amount of exoprotein below the detection limit (see Figure 1(a), Table S1 and for selected proteins Table 1). We justify this consideration because the q-OT-IT MS instrumentation is highly sensitive and has a high resolution. However, it is important to consider that the production of certain proteins, especially exoproteins, can be context dependent with respect to the biological system. However, we also present an additional heatmap (Figure 1(b)) generated after the missing values were replaced from a normal distribution following normalization using the Z-score. Both heatmaps show the similarities and differences among the four genotypes representing the four *P. larvae* ERIC strains, although each heatmap provides different benefits with respect to evaluating the data and identifying the differences. The hierarchical clustering in the columns of both heatmaps shows that the end results were similar, meaning that the ERIC I genotype was separated from the remaining three ERIC genotypes (II–IV). In addition to the two heatmap presentations (Figure 1), we performed a PCA (Figure 2) using data processed via the same algorithms. Again, both PCA charts visualize the proteome differences in the exoprotein fractions that correspond to the genetic distance among the four ERICs.

**Table 1.** Selected proteins represent the major known or novel candidate protein-derived virulence factors of the *P. larvae* ERIC I–IV genotypes. The relative mean of log2-transformed protein abundance is provided, and the white square indicates that the protein was not detected. The first UniProt protein ID (as listed in Table S1), curated protein name, description and conserved domains for the proteins are also provided. The abbreviation s.f. indicates superfamily. For the lists of UniProt protein IDs and next potential markers, see the Excel file Table S1.

Furthermore, from our analyses (Figures 1, 2), the similarity of ERICs III and IV (formerly *P. l. pulvifaciens*) [8,9] could be observed, although this was not the case for ERIC I and ERIC II (formerly *P. l. larvae*) [8,9]. We suggest that the clustering somewhat corresponds to biological observations (see [20,21]), thus demonstrating a greater ability or speed with which honeybee larva are killed by ERIC II, III and IV compared to ERIC I.

In the next sections, we discuss the known and novel candidate virulence factors of *P. larvae*. Furthermore, a qualitative/quantitative comparison is provided, and for the overall quantity, the LFQ intensity and/or MS/MS counts are used as indicators. Many identified proteins were individually examined in UniProt and NCBI and analyzed for conserved domains (CCD) [42,43] using InterPro [44] and BLASTp analyses [45]. The complete details on the LFQ identifications in each of the 28 nanoLC-MS/MS analyses, the list of protein IDs or fasta headers for each protein hit, the data evaluation results (including the statistical analysis and CCDs) are provided in Table S1. The selected proteins are provided in Table 1.

### C3larvin toxin, Plx1, Plx2 and *Pl*CBP49

C3larvin toxin (UniProt: W2E3J5) [31] and chitin-binding protein 49 (*Pl*CBP49; UniProt: J7I5J0, J7ID32) [30] were previously characterized as the major *P. larvae* virulence factors, and we provide a comparison of their protein abundances in the four type genotypes. Additionally, the Plx1 (UniProt: M9V7X5) and Plx2A/B (UniProt: M9V3B7/M9V6N3) toxins previously determined to be present in ERIC I [29], of which Plx2A has confirmed C3-like toxin activity [32], were not detected in the exoprotein fractions of the tested strains. We verified the presence of relevant sequences in our database search to confirm that the absence of Plx1 and Plx2A/B among all protein IDs in Table S1 was correct. C3larvin was identified only in the ERIC IV genotype exoprotein fraction. In contrast, *Pl*CBP49 was detected in all samples and highly expressed in ERIC II, with the lowest abundance observed in ERIC IV. Thus, it appears that *Pl*CBP49, which has confirmed chitinase activity [30], is a virulence factor utilized by all four ERICs and has the highest expression in ERIC II. In our study, *Pl*CBP49 exhibited the lowest abundance in ERIC IV, although from the known virulence factors, the expression of C3larvin in ERIC IV may compensate for the relatively low abundance of *Pl*CBP49. However, further analysis of the data revealed that additional differences in virulence were observed among the strains analyzed.

### Chitin-binding proteins, chitinases and proteins with similar modes of action *–* peptidoglycan modification

In addition to *Pl*CBP49 [30], we searched for the presence of additional virulence factors with potentially similar modes of action/activity. A chitin-binding protein detected in ERIC III has a domain architecture similar to that of *Pl*CBP49, although it has one additional domain: “ChiC_BD – Chitin-binding domain of chitinase C”. Another virulence factor that potentially attacks the peritrophic membrane is GlcNAc-binding protein A, which shares the “COG3979 – Chitodextrinase” domain with the previously mentioned proteins while also possessing a “Peptidase_M60 superfamily” domain. M60-like domains are extracellular zinc metalloproteases that target host glycoproteins involved in digestive tract colonization [46]. In support of our results, a recent study by Descamps *et al.* [47] suggested that the gbpA gene, which encodes the GlcNAc-binding protein A/Chitin binding protein, is a possible virulence factor in *P. larvae*.

Furthermore, we identified putative peptidoglycan-remodeling autolysins [48], which can possess bacteriolytic activity due to their similarity in substrate specificity to chitinases. An endo-beta-N-acetylglucosaminidase (EndoD; LytD) was highly abundant in all extracts analyzed, indicating its importance for all four genotypes, although its abundance was lowest in ERIC II. EndoD can be induced extracellularly [49] and potentially contributes to virulence and growth [50]. Next, we identified N-acetylmuramoyl-L-alanine amidase (LytC); in general, both LytD and LytC have been shown to be involved in vegetative cell separation and motility [48].

Finally, the polysaccharide deacetylases are of interest due to their potency in modifying chitin as well as petidoglycan. We identified the polysaccharide deacetylases that should be present according to CCD members of the carbohydrate esterase family 4 (CE4) [42,43]. Polysaccharide deacetylases can be important for osmotic stability and cell maintenance as shown in *Bacillus anthracis* [51,52]. However, according to the CCDs, the *P. larvae* putative polysaccharide deacetylase YheN has the same CE4_SmPgdA_like domain as *B. anthracis* Ba1977 (UniProt: A0A384LKH9); thus, a similar function in the resistance to the host lysozyme and requirement for full virulence is probable[52].

### Enolase

Enolase has been previously studied as an important *P. larvae* virulence factor that can be produced extracellularly[24]. In this study, enolase was detected in all samples and had the greatest abundance in ERIC I. Therefore, it appears that enolase is not an essential virulence factor among the ERIC genotypes, although it certainly promotes *P. larvae* pathogenicity. Specifically, enolase can be of significant importance in ERIC I, which we observed to be depleted for other important virulence factors compared to the three remaining ERICs.

### Proteases, collagenases, and peptidases

In general, proteolytic enzymes are important virulence factors in bacteria [53], and an analysis of the *P. larvae* genome suggested the presence of a number of proteases [23]. In this study, we identified a number of proteases with the potential to contribute to virulence using a proteomic approach. Most of these proteins were identified as zinc metallopeptidases, which is consistent with previously published data [25–27]. However, other families of proteolytic enzymes, such as serine, cysteine, thermophilic, carboxypeptidase, aminopeptidase and ATP-dependent enzymes, were also identified. Our results show that many of these proteases (see Table S1 for full list) were expressed across all four reference genotypes and detectable in the exoprotein fractions, with some of them exhibiting important quantitative and/or qualitative differences that indicate the potential for differences in virulence among genotypes.

We identified two candidate protease markers with excellent potential to constitute important virulence factors, the microbial collagenase ColA (ColA) and the immune inhibitor A (InhA), which were detected in ERICs II–IV and missing in ERIC I. The results also indicate that in ERIC II (Table S1), we exclusively detected the ColA isoform with 99% identity and 96% query coverage, and the second isoform, which is assigned as peptidase M9 with 99% identity and 100% query coverage. Thus, the three separate ColA hits are in fact the same protein. The detection of ColA is important because bacterial metalloproteases with collagenase activity degrade animal extracellular matrices and constitute important virulence factors [54]. InhA is an M6 family metalloprotease, and an analysis of its domain structure suggests that this protein belongs to a family of proteins called thuringilysins. These proteins have been shown to be produced by *B. thuringiensis* to eliminate insect antibacterial proteins, thereby affecting the humoral response and protecting the pathogen [55,56]. Considering that InhA is known to hydrolyze cecropins in the insect hemolymph [57], and the identification of this protein can explain the inhibition of the insect immune-associated cecropin-like activity by *P. larvae* observed by Jarosz and Glinski [33]. Our results support the previous suggestion based on a comparative genome analysis that InhA can be expressed in ERIC II but not in ERIC I [23]. Altogether, the expression pattern across the genotypes and virulence potential indicate that the ColA and InhA may be important virulence factors that cause the differences in virulence between the ERIC I and ERIC II–IV genotypes.

Additional potential protease-based virulence factors of *P. larvae* were detected as observed from the wide spectra identified (see Table S1). For instance, the putative serine protease HtrA, which was detected in all genotypes and exhibited the lowest abundance in ERIC I, was described as another important virulence factor in bacteria [58]. The cell wall endopeptidase family M23/M37 was also detected across all ERICs, although an isoform (Table S1) with 99% identity and 100% query coverage was only detected in the ERIC III and IV genotypes. Interesting virulence factors also included bacillolysin detected in ERIC IV, the putative endopeptidase LytE, Peptidase M20, and the abovementioned GlcNAc-binding protein A with the Peptidase_M60 domain. Notably, the highest abundance of some proteases identified in this study was observed in ERIC I and certain proteases were only detected in this genotype. For instance, the oligoendopeptidase pepF/M3 family, which can participate in the regulation of sporulation[59], a process that is discussed below, had the highest abundance in ERIC I.

### Novel toxin-like proteins

We discussed C3larvin in a previous section [31] and identified a set of additional probable *P. larvae* protein toxins. The first is a toxin-like protein (UniProt: A0A2L1TX76) with 64% identity and 99% query coverage to the vegetative insecticidal protein Vip1 of *B. thuringiensis* (UniProt: M1QYU2), and the domain architecture of this protein suggests that it belongs to the bacterial exotoxin B family (InterPro: IPR003896). Thus, based on our knowledge of Vip proteins [60], it can be speculated that while the Vip2 (C3larvin-like) enters the cell and exhibits ADP-ribosyltransferase activity, the Vip1 analog binds to the membrane receptors of the honeybee larval gut. Compared with C3larvin, which we detected only in ERIC IV, the putative Vip1 analog was absent in ERIC II only. However, similar abundances were observed for the remaining three genotypes. Next, the epsilon-toxin type B (UniProt: A0A2L1TZD5), which was solely detected in ERIC III and IV, can be assigned to the aerolysin-like toxin family (InterPro: IPR004991), and the domain architecture of this protein suggests that it is similar to *Clostridium* epsilon toxin ETX and *Bacillus*/*Lysinibacillus sphaericus* mosquitocidal toxin MTX2. Furthermore, we identified additional proteins assigned in UniProt as toxin-like proteins; however, we were not able to determine the CCD domains for those proteins. Moreover, these toxin-like proteins were not detected in ERIC I, although they were heterogeneously present in the other three genotypes.

Finally, ERIC III and IV produced two proteins denoted as uncharacterized (UniProt: A0A1U9YJC6 and A0A2L1U133) that had an identity of 55% and query cover of 99% between them. Importantly, both of these proteins have enterotoxin_a, RICIN and RicinB lectin domains. Interestingly, these proteins have very similar protein architectures to the mosquitocidal toxin/mosquito larvicidal toxin from *Lysinibacillus sphaericus* (MTX1; UniProt: Q03988/Q75UG7). Their similarity to MTX1 suggests that these two proteins are potential ADP-ribosylating toxins[61]. Although we observed that both the putative mosquitocidal toxins were more highly expressed in ERIC III than in ERIC IV, we detected C3larvin solely in the ERIC IV genotype. Finally, it is interesting to note that both uncharacterized proteins are similar (identity of 53% and 99% query coverage or identity of 48% and 93% query coverage, respectively) to the *P. larvae* Plx1 or toxin 1 (UniProt: M9V7X5) that was previously identified in ERIC I [29]. However, as noted above, Plx1 was not identified in our study.

### Putative bacteriocin

Previous studies have indicated that *P. larvae* produces antimicrobial compounds, such as paenilicmins and paenilarvins, which play a role in pathogenesis and are capable of influencing pathogen survival and facilitating colonization [34–36]. In this study, the detection of putative bacteriocin in all four genotypes was an interesting finding. According to its domain architecture [42,44], the putative bacteriocin (UniProt: W2EAN2) belongs to a Lactococcin 972 family (InterPro: IPR006540). If this protein has bacteriocin activity, *P. larvae* may potentially use it to compete with other bacteria [62]. The results of this study suggest that ERIC III and IV may have a greater ability to suppress other bacteria due to a higher bacteriocin abundance than was observed in ERIC I and II. Additionally, Minnaard and Alippi [63] tested the bacteriocin-like compounds of *B. cereus* towards *P. larvae*. However, our results may indicate that *P. larvae* can produce similar compounds to facilitate colonization/infection, which could be another aspect of their virulence. Previously, we showed that the microbiome can be influenced by AFB [64], and the production of such compounds can impact the microbiome.

### S-layer proteins

Other significant virulence factors include S-layer proteins. In general, S-layer proteins are important for adherence to various substrates and surfaces as well as aggregation and coaggregation, and they are also involved in protection from stressful environmental conditions [65]. A 2D-E-MS-based proteomic analysis showed that the S-layer protein A (SplA; UniProt: I0BWH5) was expressed by ERIC II but was absent in ERIC I [66], and a subsequent study supported the role of SplA [28]. In this study, we identified four S-layer proteins. In addition, the S-layer homology (SLH) domains were present in LytC and two thermophilic serine proteinases. We confirmed the presence of SplA in our dataset, and it was present in both ERIC I and II. Importantly, although there was a higher abundance in ERIC II, the SplA protein was identified in all of the biological replicates of ERIC I that we analyzed. Our results therefore suggest that SplA may be absent from ERIC III and IV, for which the other three S-layer proteins were identified. In addition, one of the S-layer proteins that was detected in ERIC III and IV has an SEC10/PgrA domain [42] in addition to the SLH domain, indicating that it may be important in surface exclusion [67,68].

### Phage proteins

Phages infecting *P. larvae* are of great importance and therefore have been isolated, and the genomes have been analyzed [69]. We identified a set of putative phage proteins (see Table S1) in the protein spectra. Moreover, the UPF0234 protein with the YajQ-like domain that was only detected in ERIC III and IV could potentially be a nucleotide-binding protein involved in the control of bacteriophage transcription [70]. Overall, our results indicate that phage proteins were identified in ERIC I, III and IV; however, the trace results also identified phage proteins in ERIC II.

### Sporulation, aggregation and cell division

There are two crucial periods in the *P. larvae* life cycle: spore germination, which occurs soon after ingestion, with the first vegetative cells visible 24 hours post infection [71]; and sporulation, which occurs during the entire infection [71] but dominates in periods of nutrient starvation and results in the production of billions of spores in each larva that can be distributed in and within colonies [72–74]. In general, only a few spores of the invasive *P. larvae* are needed to initiate the infection [75]. According to Brodsgaard et al [75], 24 to 28-hour-old larvae are the most susceptible to infection. Later, the larvae become resistant, with significant resistance observed after the larvae reached 48 hours old [75]. The identification of activators and inhibitors of spore germination has been suggested to be a useful tool for AFB control [76].

In ERIC IV, we detected the spore cortex-lytic enzyme (Table S1) likely associated with spore germination [77]. The principal function of the two penicillin-binding protein 2B (PBP-2B) isoforms (25% identity and 79% query coverage) detected in ERICs II–IV is likely vegetative cell division and the regulation of sporulation septation as observed in *Bacillus subtilis* [78]. Moreover, because a large increase in vegetative PBP-2B has been observed in *B. subtilis* during sporulation [79], these proteins are potential virulence factors associated with sporulation. Both PBP-2Bs were absent in ERIC I. The proteins identified as sporulation initiation phosphotransferase F, stage V sporulation protein T, SpoIID/LytB domain-containing protein and dipeptide-binding protein DppE function according to a GO analysis of sporulation. Moreover, members of various protease families, such as oligoendopeptidase, pepF/M3 family and oligoendopeptidase F-like protein, can participate in the sporulation process. For example, the overexpression of oligoendopeptidase F (PepF) in *B. subtilis* inhibits the process of sporulation initiation [59].

### Other proteins

We identified additional proteins that should be investigated with regards to virulence (Table S1). We identified members of the CRISPR-Cas defense system that should function against bacteriophages [80]. Potential contributors to virulence also include antioxidant enzymes, such as superoxide dismutase [81] and thioredoxin [82]. The metabolic enzyme aldehyde-alcohol dehydrogenase may control virulence as described in *Escherichia coli *[83]. Another putative virulence factor is chaperone protein DnaK [47], which exhibited no significant variation in abundance among the ERIC genotypes in this investigation. Additional groups of proteins of interest include those involved in flagellum organization since flagella play a role in adhesion and invasion to host cells [84,85]. Notably, the expression of LytC, which was discussed above, was observed to be important for flagellar function in *B. subtilis *[48]. Other potential virulence factors that are not discussed may be of interest and are listed in Table S1.

### Conclusions and future perspectives

We have shown that the label-free proteomic analysis of the *in vitro*-produced exoprotein fraction is a useful strategy for identifying the virulence factors of the dangerous honeybee pathogen *P. larvae*. The high-throughput approach provided abundant results that allowed for the identification of novel putative virulence factors and a comparison of the expression of the previously studied virulence factors among ERICs I-IV; however, certain limitations were observed. For example, the analysis was performed *in vitro* and included only one type strain per four ERIC genotypes. In the future, the key markers can be used in a targeted analysis on a wide spectrum of isolates and/or in functional studies. Based on our analysis, we suggest that the key markers are microbial collagenase ColA, InhA, different S-layer proteins and polysaccharide deacetylases, which all prevailed in ERICs II–IV. Furthermore, putative novel candidates that potentially act as toxins were identified. Interestingly, we detected a putative bacteriocin of the Lactococcin 972 family for which we hypothesize a function in competition with other bacteria. It appears that *P. larvae* shares virulence factors similar to those of the *B. cereus* group.

## Materials and methods

### Bacterial strains and growth conditions

In this study, we used the type strains of *P. larvae* obtained from the Leibniz-Institute DSMZ (https://www.dsmz.de) and the BCCM/LMG Bacteria Collection (http://bccm.belspo.be). The following strains were classified as ERIC I–IV genotypes according to Genersch *et al.* [15]: ERIC I – DSM-7030; ERIC II – DSM-25430; ERIC III – LMG-16252; and ERIC IV – DSM-3615. The bacteria were cultured at 37°C with constant shaking under aerobic conditions in MYPGP medium [86] consisting of 10 g Mueller-Hinton broth (Cat No. X927.1; Carl Roth, Karlsruhe, Germany), 15 g yeast extract (Cat No. 92144; Sigma Aldrich, St. Louis, MO, USA), 3 g K_2_HPO_4_ (Cat No. 15070–31000; Penta), 2 g glucose (Cat No. G8270; Sigma Aldrich), and 1 g sodium pyruvate (Cat No. P2256; Sigma Aldrich) per liter of medium (pH = 7.1). The bacteria were stored in 20% glycerol stocks in a freezer (SE400-906, Thermo) at −80°C until further use.

### Preparation of *paenibacillus larvae* total exoproteins

Prior to the experiment, *P. larvae* strains were precultured in 50-mL centrifuge tubes. Thirty milliliters of fresh MYPGP medium was inoculated with an appropriate amount of precultured bacteria to a starting A_595_ of 0.03–0.05 on a microplate reader Multiscan Ascent (Thermo) and cultured to the late exponential-early stationary phase. The cultivation time was as follows: ERIC I, 24 h; ERIC II, 18 h; and ERIC III and IV, 16 h. The cells were centrifuged at 6000 × g for 5 min in an MR 23i centrifuge (Jouan Industries, France), and 20 mL of each supernatant was filtered through a 0.45-μm regenerated cellulose filter (Cat No. TR-200435 OmniPeak; Teknokroma, Barcelona, Spain). The aliquots were frozen and then lyophilized in a PowerDry LL3000 lyophilizer (Thermo). The reconstituted samples were desalted and cleaned using a PD midiTrap G-25 column (Cat No. 28–9180–08; GE Healthcare Lifesciences, United Kingdom) according to the manufacturer’s instructions. The resulting cleaned supernatants were divided into appropriate aliquots for the analysis and determination of the protein content using Bradford reagent (Cat No. B6916; Sigma-Aldrich), with bovine serum albumin (Cat No. P0834; Sigma-Aldrich) used as a calibration standard. The resulting aliquots of the cleaned proteins were frozen and then lyophilized, and the samples were stored in the freezer at −80°C until use.

### Label-free proteomic analysis

The 7 biological replicates of the exoprotein fractions prepared from each ERIC genotype were used in the nanoLC-MS/MS analysis. Each lyophilizate was reconstituted in 100 mM TEAB (Cat No. 90360; Sigma-Aldrich) containing 2% SDC (Cat No. 30970; BioXtra, Sigma-Aldrich) and was heated at 95°C for 5 min. The concentration was determined using a BCA protein assay kit (Cat No. 23225; Thermo), and 25 µg of protein per sample was used for MS sample preparation. Cysteines were reduced with a 5 mM final concentration of TCEP (60°C for 60 min) and blocked with a 10 mM final concentration of MMTS (10 min at room temperature). Samples were digested with trypsin (trypsin/protein ratio 1/20) at 37°C overnight, after which the samples were acidified with trifluoroacetic acid to final concentration of 1%. SDC was removed by extraction to ethylacetate[87], and peptides were desalted on a Michrom C18 column. Dried peptides were resuspended in 25 µL of water containing 2% acetonitrile and 0.1% trifluoroacetic acid. For analysis, a 12 µL sample volume was used.

All 28 samples were consecutively analyzed using an Orbitrap^TM^ Fusion^TM^ Tribrid q-OT-IT mass spectrometer (Thermo). A reversed-phase nano column (EASY-Spray column, 50 cm × 75 µm ID, PepMap C18, 2 µm particles, 100 Å pore size) was used for the nanoLC-MS/MS analysis. The mobile phase buffer A was composed of water and 0.1% formic acid, while the mobile phase B was composed of acetonitrile and 0.1% formic acid. Samples were loaded onto the trap column (Acclaim PepMap300, C18, 5 µm, 300 Å Wide Pore, 300 µm × 5 mm) at a flow rate of 15 μL/min. The loading buffer was composed of water, 2% acetonitrile and 0.1% trifluoroacetic acid. Peptides were eluted with a gradient of B from 4 to 35% over 60 min at a flow rate of 300 nL/min. Eluted peptide cations were converted to gas-phase ions by electrospray ionization and analyzed using nanoLC-MS/MS. Survey scans of the peptide precursors from 350 to 1400 m/z were performed at a resolution of 120 K (at 200 m/z) with a 5 × 10^5^ ion count target. Tandem MS was performed by isolation at 1.5 Th with the quadrupole, HCD fragmentation with a normalized collision energy of 30, and a rapid scan MS analysis in the ion trap. The MS2 ion count target was set to 10^4^, and the max injection time was 35 ms. Only those precursors with a charge state of 2–6 were sampled for MS^2^. The dynamic exclusion duration was set to 45 s with a 10 ppm tolerance around the selected precursor and its isotopes. Monoisotopic precursor selection was turned on. The instrument was run in top speed mode with 2 s cycles [88,89].

### Data evaluation

The obtained raw data were searched and quantified with label-free algorithms using MaxQuant v1.6.3.4 [40], and the data analysis was performed using Perseus v1.6.2.3 [41]. The FDR was 1% for both proteins and peptides, and a minimum length of seven amino acids was specified. The enzyme specificity was set as C-terminal to Arg and Lys, allowing for cleavage at proline bonds and a maximum of two missed cleavages. MethylThio was set as a fixed modification, and N-terminal acetylation and methionine oxidation were the variable modifications. The database source for the identification of *P. larvae* proteins was UniProt, which contained 25,281 records of different strains when it was downloaded on 12.03.2019. To improve the protein identification and exclude contaminants from the medium, we considered the proteins that could originate from the yeast extract and Mueller-Hinton broth (contains beef fusion and casein peptone-acidic hydrolysate) in analyzing the MS data. Thus, we included 6,721 and 32,512 sequences related to *Saccharomyces cerevisiae* and *Bos taurus*, respectively, downloaded from UniProt on 12.03.2019 to the list of contaminants in MaxQuant.

The LFQ intensities were set as the main matrix. The data were log2 transformed, and the protein hits identified by site, reverse and potential contaminants were filtered out. Histograms with (Figure S1A) and without (Figure S2B) contaminants were generated. Furthermore, the data were filtered with a threshold so that they contained at least 3 valid values in at least one ERIC genotype. The groups were averaged, and the matrix was evaluated using multiple sample test (ANOVA, s0 = 0, FDR = 0.05, 250 randomizations). The missing values were replaced as follows: (a) missing values were replaced by a constant 0, or (b) missing values were replaced based on a normal distribution (width: 0.3; down shift: 1.7; total matrix). The second option was additionally normalized through the Z-score. The data were again averaged in groups and evaluated using the multiple sample test. Hierarchical clustering was performed using the average Euclidian distance (300 clusters; 10 iterations) for both row and column trees. The resulting heatmaps were adjusted to grayscale. To create the heatmap presentation in Table 1, the protein hits of interest were manually selected and the mean of the matrix with missing values replaced by the constant 0 was calculated.

## Data Availability

The accession number for the raw nanoLC-MS/MS runs reported in this paper is MassIVE MSV000083636 [doi:10.25345/C5SP8K] or PXD013272. In addition, the compressed (zipped) folder “combined”, and contaminants and *P. larvae* databases are provided.

## References

[1] Grady EN, MacDonald J, Liu L, et al Current knowledge and perspectives of *Paenibacillus*: a review. Microb Cell Fact. 2016;15:203. 27905924 10.1186/s12934-016-0603-7 PMC5134293

[2] Menendez E, Garcia-Fraile P. Plant probiotic bacteria: solutions to feed the world. AIMS Microbiol. 2017;3:502–524. 10.3934/microbiol.2017.3.502 PMC6604988 31294173

[3] Liang T-W, Wang S-L Recent advances in exopolysaccharides from *Paenibacillus* spp.: production, isolation, structure, and bioactivities. Mar Drugs. 2015;13:1847–1863. 25837984 10.3390/md13041847 PMC4413190

[4] Passera A, Marcolungo L, Casati P, et al Hybrid genome assembly and annotation of *Paenibacillus pasadenensis* strain R16 reveals insights on endophytic life style and antifungal activity. PLoS One. 2018;13:e0189993. 29351296 10.1371/journal.pone.0189993 PMC5774705

[5] Saez-Nieto JA, Medina-Pascual MJ, Carrasco G, et al *Paenibacillus* spp. isolated from human and environmental samples in Spain: detection of 11 new species. New Microbes New Infect. 2017;19:19–27. 28702198 10.1016/j.nmni.2017.05.006 PMC5484988

[6] Yousten AA Paenibacillus In: Capinera JL, editor. Encyclopedia of entomology. Dordrecht: Springer; 2018 p. 2718–2719. doi:10.1007/978-1-4020-6359-6_2724

[7] Ash C, Priest FG, Collins MD Molecular identification of rRNA group 3 bacilli (Ash, Farrow, Wallbanks and Collins) using a PCR probe test: proposal for the creation of a new genus *Paenibacillus*. Antonie Van Leeuwenhoek. 1993/1994;64:253–260. 10.1007/BF00873085 8085788

[8] Genersch E American foulbrood in honeybees and its causative agent, *Paenibacillus larvae*. J Invertebr Pathol. 2010;103:S10–9. 19909971 10.1016/j.jip.2009.06.015

[9] Heyndrickx M, Vandemeulebroecke K, Hoste B, et al Reclassification of *Paenibacillus* (formerly *Bacillus*) *pulvifaciens* (Nakamura 1984) Ash et al. 1994, a later subjective synonym of *Paenibacillus* (formerly *Bacillus*) *larvae* (white 1906) Ash et al. 1994, as a subspecies of *P. larvae*, with emended descriptions of *P. larvae* as *P. larvae* subsp. *larvae* and *P. larvae* subsp. *pulvifaciens*. Int J Syst Bacteriol. 1996;46:270–279. 8573507 10.1099/00207713-46-1-270

[10] Nakamura LK *Bacillus pulvifaciens* sp. nov., nom. rev. Int J Syst Bacteriol. 1984;34:410–413. 10.1099/00207713-40-3-242 2397192

[11] Versalovic J, Schneider M, De Bruijn FJ, et al Genomic fingerprinting of bacteria using repetitive sequence-based polymerase chain reaction. Methods Mol Cell Biol. 1994;5:25–40.

[12] Genersch E, Otten C The use of repetitive element PCR fingerprinting (rep-PCR) for genetic subtyping of German field isolates of *Paenibacillus larvae* subsp. *larvae*. Apidologie. 2003;34:195–206.

[13] Neuendorf S, Hedtke K, Tangen G, et al Biochemical characterization of different genotypes of *Paenibacillus larvae* subsp. *larvae*, a honey bee bacterial pathogen. Microbiology. 2004;150:2381–2390. 15256579 10.1099/mic.0.27125-0

[14] Genersch E, Ashiralieva A, Fries I Strain- and genotype-specific differences in virulence of *Paenibacillus larvae* subsp. *larvae*, a bacterial pathogen causing American foulbrood disease in honeybees. Appl Environ Microbiol. 2005;71:7551–7555. 16269801 10.1128/AEM.71.11.7551-7555.2005 PMC1287710

[15] Genersch E, Forsgren E, Pentikainen J, et al Reclassiﬁcation of *Paenibacillus larvae* subsp. *pulvifaciens* and *Paenibacillus larva*e subsp. *larvae* as *Paenibacillus larvae* without subspecies differentiation. Int J Syst Evol Microbiol. 2006;56:501–511. 16514018 10.1099/ijs.0.63928-0

[16] Schafer MO, Genersch E, Funfhaus A, et al Rapid identification of differentially virulent genotypes of *Paenibacillus larvae*, the causative organism of American foulbrood of honey bees, by whole cell MALDI-TOF mass spectrometry. Vet Microbiol. 2014;170:291–297. 24613082 10.1016/j.vetmic.2014.02.006

[17] Morrissey BJ, Helgason T, Poppinga L, et al Biogeography of *Paenibacillus larvae*, the causative agent of American foulbrood, using a new multilocus sequence typing scheme. Environ Microbiol. 2015;17:1414–1424. 25244044 10.1111/1462-2920.12625 PMC4405054

[18] Descamps T, De Smet L, Stragier P, et al Multiple locus variable number of tandem repeat analysis: a molecular genotyping tool for *Paenibacillus larvae*. Microb Biotechnol. 2016;9:772–781. 27365124 10.1111/1751-7915.12375 PMC5072193

[19] Poppinga L, Genersch E Molecular pathogenesis of American foulbrood: how *Paenibacillus larvae* kills honey bee larvae. Curr Opin Insect Sci. 2015;10:29–36. 29588011 10.1016/j.cois.2015.04.013

[20] Ashiralieva A, Genersch E Reclassification, genotypes and virulence of *Paenibacillus larvae*, the etiological agent of American foulbrood in honeybees – a review. Apidologie. 2006;37:411–420.

[21] Rauch S, Ashiralieva A, Hedtke K, et al Negative correlation between individual-insect-level virulence and colony-level virulence of *Paenibacillus larvae*, the etiological agent of American foulbrood of honeybees. Appl Environ Microbiol. 2009;75:3344–3347. 19304833 10.1128/AEM.02839-08 PMC2681656

[22] Hitchcock JD, Stoner A, Wilson WT, et al Pathogenicity of *Bacillus pulvifaciens* to honey bee larvae of various ages (Hymenoptera: apidae). J Kansas Entomol Soc. 1979;52:238–246.

[23] Djukic M, Brzuszkiewicz E, Funfhaus A, et al How to kill the honey bee larva: genomic potential and virulence mechanisms of *Paenibacillus larvae*. PLoS One. 2014;9:e90914. 24599066 10.1371/journal.pone.0090914 PMC3944939

[24] Antunez K, Anido M, Arredondo D, et al *Paenibacillus larvae* enolase as a virulence factor in honeybee larvae infection. Vet Microbiol. 2011;147:83–89. 20609532 10.1016/j.vetmic.2010.06.004

[25] Antunez K, Arredondo D, Anido M, et al Metalloprotease production by *Paenibacillus larvae* during the infection of honeybee larvae. Microbiology. 2011;157:1474–1480. 21330433 10.1099/mic.0.044321-0

[26] Hrabak J, Martinek K Screening of secreted proteases of *Paenibacillus larvae* by using substrate-SDS-polyacrylamide gel electrophoresis. J Apic Res Bee World. 2007;46:160–164.

[27] Dancer BN, Chantawannakul P The proteases of American foulbrood scales. J Invertebr Pathol. 1997;70:79–87. 9281394 10.1006/jipa.1997.4672

[28] Poppinga L, Janesch B, Funfhaus A, et al Identification and functional analysis of the S-layer protein SplA of *Paenibacillus larvae*, the causative agent of American foulbrood of honey bees. PLoS Pathog. 2012;8:e1002716. 22615573 10.1371/journal.ppat.1002716 PMC3355101

[29] Funfhaus A, Poppinga L, Genersch E Identification and characterization of two novel toxins expressed by the lethal honey bee pathogen *Paenibacillus larvae*, the causative agent of American foulbrood. Environ Microbiol. 2013;15:2951–2965. 23992535 10.1111/1462-2920.12229

[30] Garcia-Gonzalez E, Poppinga L, Funfhaus A, et al *Paenibacillus larvae* chitin-degrading protein *Pl*CBP49 is a key virulence factor in American foulbrood of honey bees. PLoS Pathog. 2014;10:e1004284. 25080221 10.1371/journal.ppat.1004284 PMC4117609

[31] Krska D, Ravulapalli R, Fieldhouse RJ, et al C3larvin toxin, an ADP-ribosyltransferase from *Paenibacillus larvae*. J Biol Chem. 2015;290:1639–1653. 25477523 10.1074/jbc.M114.589846 PMC4340408

[32] Ebeling J, Funfhaus A, Knispel H, et al Characterization of the toxin Plx2A, a RhoA‐targeting ADP‐ribosyltransferase produced by the honey bee pathogen *Paenibacillus larvae*. Environ Microbiol. 2017;19:5100–5116. 29124866 10.1111/1462-2920.13989

[33] Jarosz J, Glinski Z Selective inhibition of cecropin-like activity of insect immune blood by protease from American foulbrood scales. J Invertebr Pathol. 1990;56:143–149. 2273283 10.1016/0022-2011(90)90096-o

[34] Muller S, Garcia-Gonzalez E, Mainz A, et al Paenilamicin: structure and biosynthesis of a hybrid nonribosomal peptide/polyketide antibiotic from the bee pathogen *Paenibacillus larvae*. Angew Chem Int Ed Engl. 2014;53:10821–10825. 25080172 10.1002/anie.201404572

[35] Hertlein G, Seiffert M, Gensel S, et al Biological role of paenilarvins, iturin-like lipopeptide secondary metabolites produced by the honey bee pathogen *Paenibacillus larvae*. PLoS One. 2016;11:e0164656. 27760211 10.1371/journal.pone.0164656 PMC5070912

[36] Sood S, Steinmetz H, Beims H, et al Paenilarvins: iturin family lipopeptides from the honey bee pathogen *Paenibacillus larvae*. ChemBioChem. 2014;15:1947–1955. 25069424 10.1002/cbic.201402139

[37] Hertlein G, Muller S, Garcia-Gonzalez E, et al Production of the catechol type siderophore bacillibactin by the honey bee pathogen *Paenibacillus larvae*. PLoS One. 2014;9:e108272. 25237888 10.1371/journal.pone.0108272 PMC4169593

[38] Schild H-A, Fuchs SW, Bode HB, et al Low-molecular-weight metabolites secreted by *Paenibacillus larvae* as potential virulence factors of American foulbrood. Appl Environ Microbiol. 2014;80:2484–2492. 24509920 10.1128/AEM.04049-13 PMC3993163

[39] Garcia-Gonzalez E, Muller S, Hertlein G, et al Biological effects of paenilamicin, a secondary metabolite antibiotic produced by the honey bee pathogenic bacterium *Paenibacillus larvae*. MicrobiologyOpen. 2014;3:642–656. 25044543 10.1002/mbo3.195 PMC4234257

[40] Cox J, Hein MY, Luber CA, et al Accurate proteome-wide label-free quantification by delayed normalization and maximal peptide ratio extraction, termed MaxLFQ. Mol Cell Proteomics. 2014;13:2513–2526. 24942700 10.1074/mcp.M113.031591 PMC4159666

[41] Tyanova S, Temu T, Sinitcyn P, et al The Perseus computational platform for comprehensive analysis of (prote)omics data. Nat Methods. 2016;13:731–740. 27348712 10.1038/nmeth.3901

[42] Geer LY, Domrachev M, Lipman DJ, et al CDART: protein homology by domain architecture. Genome Res. 2002;12:1619–1623. 12368255 10.1101/gr.278202 PMC187533

[43] Marchler-Bauer A, Bo Y, Han L, et al CDD/SPARCLE: functional classification of proteins via subfamily domain architectures. Nucleic Acids Res. 2017;45:D200–3. 27899674 10.1093/nar/gkw1129 PMC5210587

[44] Finn RD, Attwood TK, Babbitt PC, et al InterPro in 2017—beyond protein family and domain annotations. Nucleic Acids Res. 2017;45:D190–9. 27899635 10.1093/nar/gkw1107 PMC5210578

[45] Altschul SF, Gish W, Miller W, et al Basic local alignment search tool. J Mol Biol. 1990;215:403–410. 2231712 10.1016/S0022-2836(05)80360-2

[46] Nakjang S, Ndeh DA, Wipat A, et al A novel extracellular metallopeptidase domain shared by animal host-associated mutualistic and pathogenic microbes. PLoS One. 2012;7:e30287. 22299034 10.1371/journal.pone.0030287 PMC3267712

[47] Descamps T, De Smet L, De Vos P, et al Unbiased random mutagenesis contributes to a better understanding of the virulent behaviour of *Paenibacillus larvae*. J Appl Microbiol. 2018;124:28–41. 29044873 10.1111/jam.13611

[48] Chen R, Guttenplan SB, Blair KM, et al Role of the σ^D^-dependent autolysins in *Bacillus subtilis* population heterogeneity. J Bacteriol. 2009;191:5775–5784. 19542270 10.1128/JB.00521-09 PMC2737971

[49] Takegawa K, Nakoshi M, Iwahara S, et al Induction and purification of endo-β-*N*-acetylglucosaminidase from *Arthrobacter protophormiae* grown in ovalbumin. Appl Environ Microbiol. 1989;55:3107–3112. 16348072 10.1128/aem.55.12.3107-3112.1989 PMC203231

[50] Robb M, Hobbs JK, Woodiga SA, Shapiro-Ward S, Suits MDL, McGregor N, Brumer H, Yesilkaya H, King SJ, Boraston AB Molecular characterization of N-glycan degradation and transport in *Streptococcus pneumoniae* and its contribution to virulence. PLoS Pathog. 2017;13:e1006090. 28056108 10.1371/journal.ppat.1006090 PMC5215778

[51] Arnaouteli S, Giastas P, Andreou A, et al Two putative polysaccharide deacetylases are required for osmotic stability and cell shape maintenance in *Bacillus anthracis*. J Biol Chem. 2015;290:13465–13478. 25825488 10.1074/jbc.M115.640029 PMC4505593

[52] Balomenou S, Fouet A, Tzanodaskalaki M, et al Distinct functions of polysaccharide deacetylases in cell shape, neutral polysaccharide synthesis and virulence of *Bacillus anthracis*. Mol Microbiol. 2013;87:867–883. 23336745 10.1111/mmi.12137

[53] Lebrun I, Marques-Porto R, Pereira AS, et al Bacterial toxins: an overview on bacterial proteases and their action as virulence factors. Mini Rev Med Chem. 2009;9:820–828. 19519507 10.2174/138955709788452603

[54] Duarte AS, Correia A, Esteves AC Bacterial collagenases – a review. Crit Rev Microbiol. 2016;42:106–126. 24754251 10.3109/1040841X.2014.904270

[55] Edlund T, Siden I, Boman HG Evidence for two immune inhibitors from *Bacillus thuringiensis* interfering with the humoral defense system of saturniid pupae. Infect Immun. 1976;14:934–941. 992874 10.1128/iai.14.4.934-941.1976 PMC415474

[56] Lovgren A, Zhang M, Engstrom A, et al Molecular characterization of immune inhibitor A, a secreted virulence protease from *Bacillus thuringiensis*. Mol Microbiol. 1990;4:2137–2146. 2089225 10.1111/j.1365-2958.1990.tb00575.x

[57] Dalhammar G, Steiner H Characterization of inhibitor A, a protease from *Bacillus thuringiensis* which degrades attacins and cecropins, two classes of antibacterial proteins in insects. Eur J Biochem. 1984;139:247–252. 6421577 10.1111/j.1432-1033.1984.tb08000.x

[58] de Stoppelaar SF, Bootsma HJ, Zomer A, et al *Streptococcus pneumoniae* serine protease HtrA, but not SFP or PrtA, is a major virulence factor in pneumonia. PLoS One. 2013;8:e80062. 24244609 10.1371/journal.pone.0080062 PMC3823867

[59] Kanamaru K, Stephenson S, Perego M Overexpression of the PepF oligopeptidase inhibits sporulation initiation in *Bacillus subtilis*. J Bacteriol. 2002;184:43–50. 11741842 10.1128/JB.184.1.43-50.2002 PMC134765

[60] Chakroun M, Banyuls N, Bel Y, et al Bacterial vegetative insecticidal proteins (Vip) from entomopathogenic bacteria. Microbiol Mol Biol Rev. 2016;80:329–350. 26935135 10.1128/MMBR.00060-15 PMC4867366

[61] Reinert DJ, Carpusca I, Aktories K, et al Structure of the mosquitocidal toxin from *Bacillus sphaericus*. J Mol Biol. 2006;357:1226–1236. 16483607 10.1016/j.jmb.2006.01.025

[62] Martinez B, Fernandez M, Suarez JE, et al Synthesis of lactococcin 972, a bacteriocin produced by *Lactococcus lactis* IPLA 972, depends on the expression of a plasmid-encoded bicistronic operon. Microbiology. 1999;145:3155–3161. 10589723 10.1099/00221287-145-11-3155

[63] Minnaard J, Alippi AM Partial characterization of bacteriocin-like compounds from two strains of *Bacillus cereus* with biological activity against *Paenibacillus larvae*, the causal agent of American foulbrood disease. Lett Appl Microbiol. 2016;63:442–449. 27589675 10.1111/lam.12665

[64] Erban T, Ledvinka O, Kamler M, et al (*Apis mellifera*)-associated bacterial community affected by American foulbrood: detection of *Paenibacillus larvae* via microbiome analysis. Sci Rep. 2017;7:5084. 28698604 10.1038/s41598-017-05076-8 PMC5506040

[65] Gerbino E, Carasi P, Mobili P, et al Role of S-layer proteins in bacteria. World J Microbiol Biotechnol. 2015;31:1877–1887. 26410425 10.1007/s11274-015-1952-9

[66] Funfhaus A, Genersch E Proteome analysis of *Paenibacillus larvae* reveals the existence of a putative S-layer protein. Environ Microbiol Rep. 2012;4:194–202. 23757273 10.1111/j.1758-2229.2011.00320.x

[67] Dunny GM, Zimmerman DL, Tortorello ML Induction of surface exclusion (entry exclusion) by *Streptococcus faecalis* sex pheromones: use of monoclonal antibodies to identify an inducible surface antigen involved in the exclusion process. Proc Natl Acad Sci U S A. 1985;82:8582–8586. 3936037 10.1073/pnas.82.24.8582 PMC390961

[68] Kao SM, Olmsted SB, Viksnins AS, et al Molecular and genetic analysis of a region of plasmid pCF10 containing positive control genes and structural genes encoding surface proteins involved in pheromone-inducible conjugation in *Enterococcus faecalis*. J Bacteriol. 1991;173:7650–7664. 1938961 10.1128/jb.173.23.7650-7664.1991 PMC212534

[69] Stamereilers C, Fajardo CP, Walker JK, et al Genomic analysis of 48 *Paenibacillus larvae* bacteriophages. Viruses. 2018;10:E377. 30029517 10.3390/v10070377 PMC6070908

[70] Qiao X, Sun Y, Qiao J, et al The role of host protein YajQ in the temporal control of transcription in bacteriophage Φ6. Proc Natl Acad Sci U S A. 2008;105:15956–15960. 18836083 10.1073/pnas.0807489105 PMC2572959

[71] Yue D, Nordhoff M, Wieler LH, et al *in situ* hybridization (FISH) analysis of the interactions between honeybee larvae and *Paenibacillus larvae*, the causative agent of American foulbrood of honeybees (*Apis mellifera*). Environ Microbiol. 2008;10:1612–1620. 18331334 10.1111/j.1462-2920.2008.01579.x

[72] Lindstrom A, Korpela S, Fries I The distribution of *Paenibacillus larva*e spores in adult bees and honey and larval mortality, following the addition of American foulbrood diseased brood or spore-contaminated honey in honey bee (*Apis mellifera*) colonies. J Invertebr Pathol. 2008;99:82–86. 18640122 10.1016/j.jip.2008.06.010

[73] Fries I, Camazine S Implications of horizontal and vertical pathogen transmission for honey bee epidemiology. Apidologie. 2001;32:199–214.

[74] Gillard M, Charriere JD, Belloy L Distribution of *Paenibacillus larvae* spores inside honey bee colonies and its relevance for diagnosis. J Invertebr Pathol. 2008;99:92–95. 18573258 10.1016/j.jip.2008.05.010

[75] Brodsgaard CJ, Ritter W, Hansen H Response of *in vitro* reared honey bee larvae to various doses of *Paenibacillus larvae larvae* spores. Apidologie. 1998;29:569–578.

[76] Alvarado I, Phui A, Elekonich MM, et al Requirements for *in vitro* germination of *Paenibacillus larvae* spores. J Bacteriol. 2013;195:1005–1011. 23264573 10.1128/JB.01958-12 PMC3571325

[77] Chirakkal H, O’Rourke M, Atrih A, et al Analysis of spore cortex lytic enzymes and related proteins in *Bacillus subtilis* endospore germination. Microbiology. 2002;148:2383–2392. 12177332 10.1099/00221287-148-8-2383

[78] Daniel RA, Harry EJ, Errington J Role of penicillin-binding protein PBP 2B in assembly and functioning of the division machinery of *Bacillus subtilis*. Mol Microbiol. 2000;35:299–311. 10652091 10.1046/j.1365-2958.2000.01724.x

[79] Sowell MO, Buchanan CE Changes in penicillin-binding proteins during sporulation of *Bacillus subtilis*. J Bacteriol. 1983;153:1331–1337. 6402492 10.1128/jb.153.3.1331-1337.1983 PMC221781

[80] Barrangou R, Fremaux C, Deveau H, et al CRISPR provides acquired resistance against viruses in prokaryotes. Science. 2007;315:1709–1712. 17379808 10.1126/science.1138140

[81] Langford PR, Sansone A, Valenti P, et al Bacterial superoxide dismutase and virulence. Method Enzymol. 2002;349:155–166. 10.1016/s0076-6879(02)49331-7 11912905

[82] Bjur E, Eriksson-Ygberg S, Aslund F, et al 1 promotes intracellular replication and virulence of *Salmonella enterica* serovar *Typhimurium*. Infect Immun. 2006;74:5140–5151. 16926406 10.1128/IAI.00449-06 PMC1594827

[83] Beckham KSH, Connolly JPR, Ritchie JM, et al The metabolic enzyme AdhE controls the virulence of *Escherichia coli* O157:H7. Mol Microbiol. 2014;93:199–211. 24846743 10.1111/mmi.12651 PMC4249723

[84] Qwt C, Rs C, Birol I, et al Updated genome assembly and annotation of *Paenibacillus larvae*, the agent of American foulbrood disease of honey bees. BMC Genomics. 2011;12:450. 21923906 10.1186/1471-2164-12-450 PMC3188533

[85] Haiko J, Westerlund-Wikstrom B The role of the bacterial flagellum in adhesion and virulence. Biology (Basel). 2013;2:1242–1267. 24833223 10.3390/biology2041242 PMC4009794

[86] Dingman DW, Stahly DP Medium promoting sporulation of *Bacillus larvae* and metabolism of medium components. Appl Environ Microbiol. 1983;46:860–869. 16346399 10.1128/aem.46.4.860-869.1983 PMC239480

[87] Masuda T, Tomita M, Ishihama Y Phase transfer surfactant-aided trypsin digestion for membrane proteome analysis. J Proteome Res. 2008;7:731–740. 18183947 10.1021/pr700658q

[88] Hebert AS, Richards AL, Bailey DJ, et al The one hour yeast proteome. Mol Cell Proteomics. 2014;13:339–347. 24143002 10.1074/mcp.M113.034769 PMC3879625

[89] Erban T, Harant K, Chalupnikova J, et al Beyond the survival and death of the deltamethrin-threatened pollen beetle *Meligethes aeneus*: an in-depth proteomic study employing a transcriptome database. J Proteomics. 2017;150:281–289. 27705816 10.1016/j.jprot.2016.09.016

