# Supplementary material for: Comprehensive proteomic analysis of exoproteins expressed by ERIC I, II, III and IV Paenibacillus larvae genotypes reveals a wide range of virulence factors
Source: Virulence. 2019 Apr 18;10(1):363–75. doi: 10.1080/21505594.2019.1603133 (PMC6527061; doi:10.1080/21505594.2019.1603133)

Fig. S1. Histograms A) with and B) without contaminants of each of the 28 nanoLC-MS/MS runs. The contaminants database consisted of common contaminants included in MaxQuant and the yeast and bovine sequences included in this work to prevent incorrect identifications related to proteins of cultivation media.

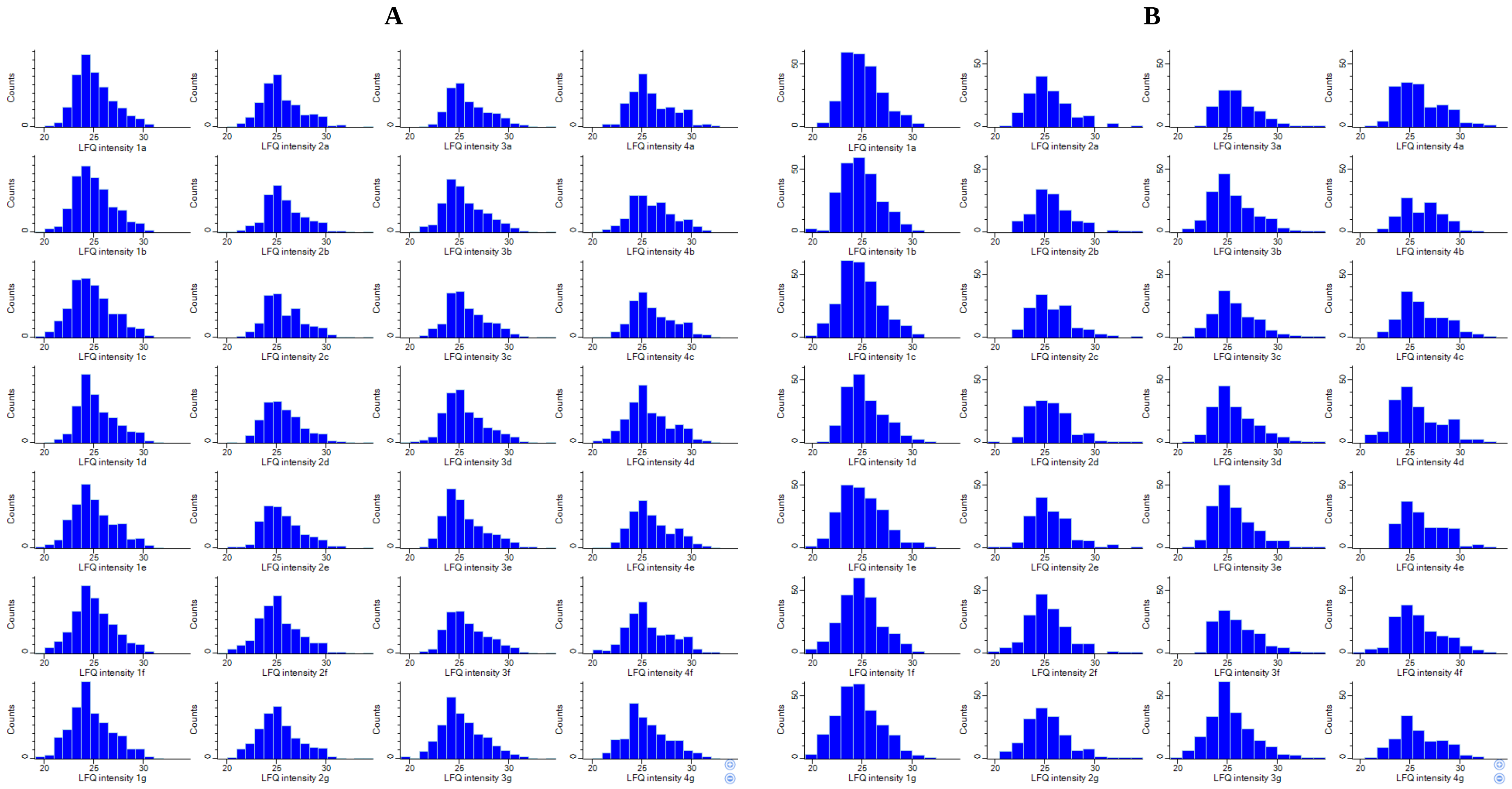

Supplement: Supplemental Material [file kvir-10-01-1603133-s001.pdf]
